# Cost-effectiveness of fixed-dose combination pill (Polypill) in primary and secondary prevention of cardiovascular disease: A systematic literature review

**DOI:** 10.1371/journal.pone.0271908

**Published:** 2022-07-28

**Authors:** Reza Jahangiri, Aziz Rezapour, Reza Malekzadeh, Alireza Olyaeemanesh, Gholamreza Roshandel, Seyed Abbas Motevalian

**Affiliations:** 1 Department of Health Economics, School of Health Management and Information Sciences, Iran University of Medical Sciences, Tehran, Iran; 2 Health Management and Economics Research Center, School of Health Management and Information Sciences, Iran University of Medical Sciences, Tehran, Iran; 3 Digestive Diseases Research Center, Digestive Diseases Research Institute, Tehran University of Medical Sciences, Tehran, Iran; 4 National Institute for Health Research & Health Equity Research Centre, Tehran University of Medical Sciences, Tehran, Iran; 5 Golestan Research Center of Gastroenterology and Hepatology, Golestan University of Medical Sciences, Gorgan, Iran; 6 Department of Epidemiology, School of Public Health, Iran University of Medical Sciences, Tehran, Iran; University of Waterloo, CANADA

## Abstract

**Background:**

A significant proportion of cardiovascular disease (CVD) morbidity and mortality could be prevented via the population-based and cost-effective interventions. A fixed-dose combination treatment is known as the polypill for the primary and secondary prevention of CVD has come up in recent years.

**Purpose:**

In order to provide recommendations for future economic evaluations, this systematic review aimed to review and assess the quality of published evidence on the cost-effectiveness of polypill in primary and secondary prevention of CVD, to identify the key drivers that impact the cost-effectiveness

**Methods:**

A systematic review of literature, following the PRISMA guidelines, was undertaken in the electronic databases. Two researchers identified the relevant studies according to inclusion and exclusion criteria. Consolidated Health Economic Evaluation Reporting Standards (CHEERS) checklist was used to quality assessment of included studies. ICERs value adjusted to 2020 United States Dollar using consumer price index (CPI) and purchasing power parity (PPP). Finally, data were summarized via a narrative synthesis.

**Results:**

In total, 24 articles were identified based on the determined inclusion criteria. All studies met more than 50% of the CHEERS criteria. Adjusted incremental cost-effectiveness ratios varied from 24$ to 31000$(2020 US dollar) among the studies. The polypill resulted in the improved adherence and quality of life, at a price equal to or lower than multiple monotherapies. This price is typically below the commonly accepted thresholds or cost saving in both, primary and secondary prevention of CVD. The main identified cost-effectiveness drivers were the polypill price, adherence, age, CVD risk, and drug combination.

**Conclusions:**

This systematic review found that the polypill seemed to be a cost-effective intervention in primary and secondary prevention of CVD. However, it is necessary to conduct more economic evaluation studies based on the long-term clinical trials with large populations. Also, studies should consider how the polypill interacts with other primary and secondary preventive strategies as a complementary health strategy.

## Introduction

Cardiovascular diseases (CVD) are the leading cause of morbidity and mortality worldwide. According to World Health Organization more than 17 million deaths from CVD occur worldwide each year [1]. Demographic changes and population growth, increasing urbanization, as well as changing behaviors and lifestyles are exacerbating this trend [2, 3]. The effects of CVD are not limited to mortality and disability. These effects also have important economic consequences. This economic burden is due to the cost of treatment and reduced productivity [4, 5].

Although CVD imposes a considerable economic burden on healthcare systems it is among the most preventable health problems [6]. Usually, interventions which delay the onset of a disease are defined as primary prevention and those which delay the progression of disease by treatment and rehabilitation are defined as secondary prevention [7]. Primary and secondary prevention of CVD by reducing and controlling some modifiable risk factors such as blood pressure and cholesterol will considerably reduce the incidence of cardiovascular events in high-risk individuals as well as cardiovascular patients [8]. Among these, drug therapy by the combined use of aspirin, statins, and antihypertensive drugs, is one of the most effective methods of prevention in high-risk individuals (primary prevention) and cardiovascular patients (secondary prevention) [9].

Non-adherence to treatment because of the multiplicity and unavailability, under-prescription and unaffordability of drugs are the most important factors in the lack of optimal implementation of primary and secondary prevention [10, 11]. Regarding the above-mentioned barriers and multiplicity of CVD prevention drugs; a fixed combination of multiple drugs in a single tablet or capsule (polypill) may reduce these barriers in the long run [12, 13]. The concept of polypill was first introduced in 2003 by Nicholas Wald and Malcolm Law [14, 15]. It is a combination of two or more medications, including the antihypertensive drugs from different classes, aspirin, statin, and folic acid for high risk people at as well as cardiovascular patients [10]. Various studies have reported the effectiveness of polypill strategy as an alternative option to improve the clinical status and adherence to treatment in the primary and secondary prevention of CVD [16–22].

In policymaking, in addition to considering the clinical efficacy of a prevention strategy, it is necessary to evaluate the cost-effectiveness aspects [23]. Cost-effectiveness analysis of polypill plays an important role in determining drug coverage, reimbursement, and decision-making optimal allocation of limited financial resources of the health system. Several primary economic evaluation studies in different countries showed that polypill strategies can be cost-effective in primary and secondary prevention [24–28]. However, there are contradictory findings regarding the price at which the polypill is cost-effective, [24, 29] correct indications and subgroups [24, 27, 29] as well as the proper composition of the drugs in polypill [24, 25, 29, 30]. So, the current study tried to assess the cost effectiveness of using polypill strategy compared to usual care in the primary and secondary prevention of CVD.

## Materials and methods

### Systematic literature search

A systematic review of literature, following the Preferred Reporting Items for Systematic Review and Meta-Analyses (PRISMA) guidelines [31], on the economic evaluation of the polypill for primary and secondary prevention of CVD was undertaken (S1 Table). An electronic literature search was conducted in PubMed/MEDLINE, Embase, Web of Science, EconLit, CINAHL, Scopus, and Cochrane Library electronic databases from January 2003 (the first time that the polypill was recommended as a prevention strategy) to December 2020. For grey literature, Google, Open Gray, the database of the World Health Organization, and World Bank website were also searched. To further complement of database search, the reference lists of the included articles pursued. Studies were identified using the following search terms, which were combined: “Economic evaluation “, “cost-effectiveness analysis”, “Cost utility analysis”, “Cost benefit analysis”, “primary prevention”, "secondary prevention", "Cardiovascular disease", "heart disease", "Myocardial infraction", Polypill, “Fixed dose combination" and “Multidrug”. Also, detailed individual search strategies were developed for each of the databases (S2 Table).

This review was registered in PROSPERO International prospective register of systematic reviews (registration number: CRD42016043510) at the Centre for Reviews and Dissemination, University of York, UK [32].

### Study selection

Studies identified from the searches were imported to the EndNote, and duplicates were removed. To meet the inclusion criteria, the studies were reviewed based on the PICOS (Population, Intervention, Comparator, Outcomes, and Study design) framework. Titles and abstracts of identified studies were being checked by two investigators. Only full economic evaluations (cost-effectiveness analysis (CEA), cost-benefit analysis (CBA), or cost-utility analyses (CUA)) were considered in the review if they focused on the primary or secondary prevention of the CVD using a polypill. Studies were excluded from the review if they were partial economic evaluations which measure only costs of an intervention without comparator (i.e. cost analyses, cost-description studies, cost-outcome descriptions, cost minimization studies), narrative reviews, letters to the editor, case series, and others lacking explicit methods. The full text of all retrieved potentially eligible studies was independently assessed against the eligibility criteria by two investigators. Any disagreements were be resolved by referral to a third member of the research team.

### Data extraction

For the data extraction from the final included articles, a standardized form was developed for this research. The extraction form included the following information: first author’s name; publication year; country; study design; type of prevention; study perspective; model type; Time horizon; intervention, comparator; effectiveness unit; incremental cost-effectiveness ratios (ICERs); sensitivity analyses; discount rate and threshold. Data extraction was carried out by one investigator and checked by another investigator.

### Quality assessment

The quality of reporting in each included research was assessed using the Consolidated Health Economic Evaluation Reporting Standards (CHEERS) statement checklist [33]. This checklist was created to examine the adequacy of the modeling methodologies and structures, the quality of reporting, and any restrictions that may have harmed the research results’ validity and generalizability. This instrument consists of a 24-item checklist verifying the presence of specific issues (e.g., perspective, comparators, and time horizon) in the considered papers. Two authors reviewed the studies and a percentage score for each study was calculated. Then the studies were categorized based on these scores. A study was deemed to be of excellent reporting quality if it scored 85% or higher, very good quality if it scored 75–85%, good quality if it scored 50–75%, and studies scoring below 50% were classified as poor quality [34, 35].

### Analysis

Studies were reviewed via a narrative synthesis with full tabulation of the results of all included studies. In order to facilitate comparisons, the ICERs value obtained in different studies, firstly was inflated to 2020 prices, using consumer price index (CPI) of each country, and then their variances in each currency were standardized by converting to 2020 United States Dollar (USD) using purchasing power parity (PPP) [36]. Based on the recommendations from guidelines for systematic reviews in economic evaluations, no attempts were made to quantitatively pool the results of the included studies [37].

## Results

### Review search results

**Fig 1:**
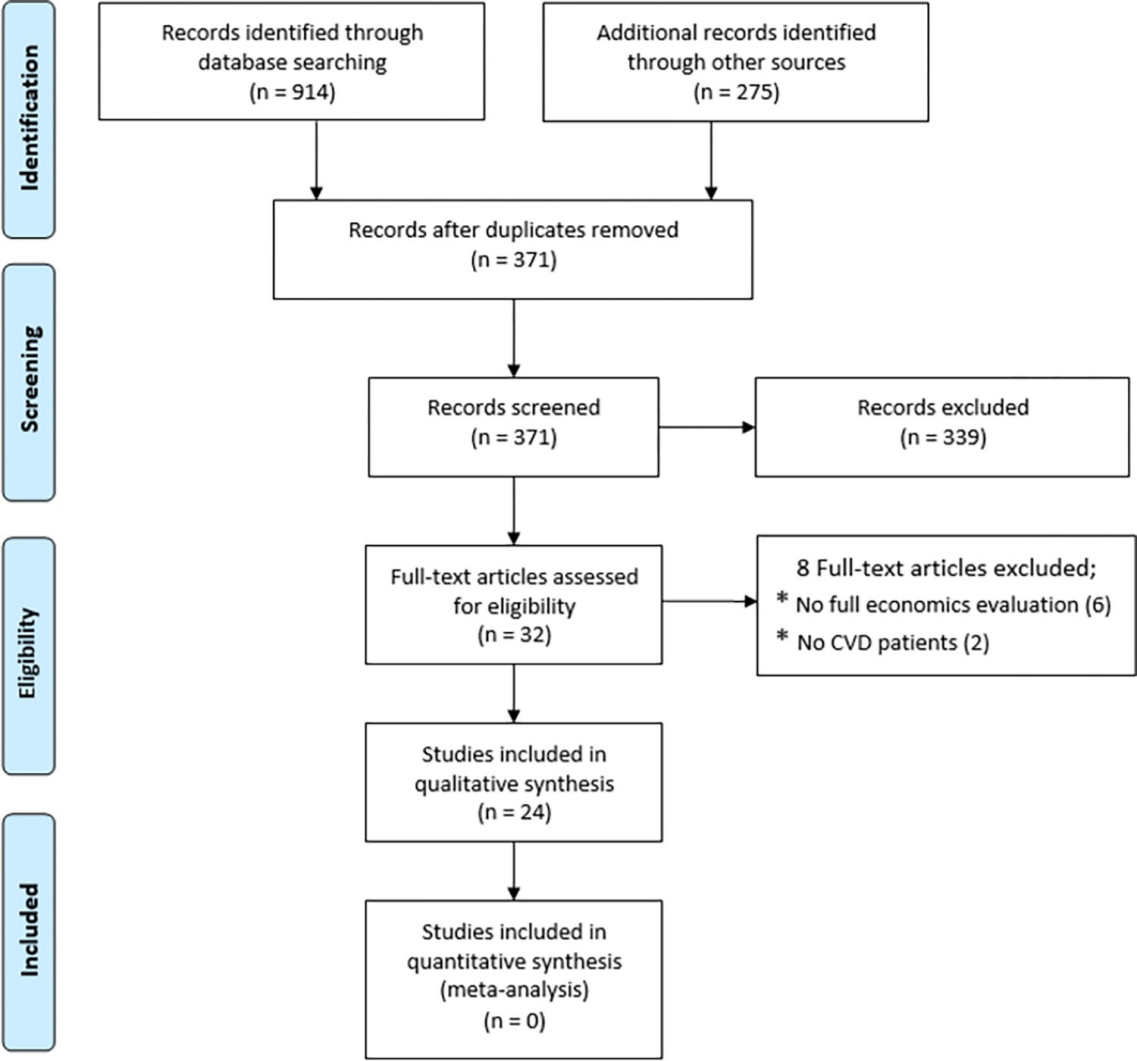
PRISMA flow diagram for study selection.

In total, 371 articles were identified from the literature search. After removing duplicates, titles and abstracts were screened for potentially relevant studies. After removing irrelevant articles, 32 studies remained for full-text examination, and 24 references met the selection criteria involved in the data extraction and quality assessment. Fig 1 shows the searching, screening and inclusion process that is summarized in the PRISMA flowchart.

### General characteristics of the included studies

Studies were published from 2006 to 2019, and nearly half of them (11, 46%) were published in the years 2014–2019. The majority of the investigations were conducted in European nations, with three studies conducted in Asia. In addition, four multi-country studies were conducted. Polypill has been investigated in 12 studies as a primary strategy, ten studies as a secondary prevention strategy, and two studies as a combined strategy. With the exception of one study [26], which used CBA, all other research used CEA. The Markov model was employed in the majority of research, with two studies using micro-simulation [24, 38] and one used within-trial cost-effectiveness analysis [29]. The type of model in Rubinstein et al [39] and Wald et al [26] is unclear.

The majority of cost-effectiveness analyses were conducted using a life time (12, 50%) and 10 years (9, 37%) time horizon. QALY was presented as the effectiveness measure in most of publications (14, 58%). Three-quarters of the studies analyzed from a healthcare perspective include direct costs associated with the intervention and the disease. A societal perspective that also captures indirect costs, such as productivity losses has been used in only two studies. More details on the general characteristics of final articles are presented in Table 1.

**Table 1:** General characteristics of the included studies.

First author & Publication year	Country	Study design	Type of prevention	Perspective	Model type	Time horizon	intervention	Comparator	Effectiveness Unit	Original ICER	Sensitivity Analysis	Discount rate	Threshold	Adjusted ICER (2020 US dollars)
**Franco et al (2006) [40]**	Netherlands	CEA	primary	Payer	Markov	10-years	Polypill (a statin, three blood pressure lowering, folic acid, aspirin)	Aspirin	YLs	Non	DSA PSA	4%	30,000	Non
**Gaziano et al (2006) [6]**	Low and middle income countries	CEA	Primary & Secondary	Health care	Markov	life time	Polypill (81 mg aspirin, 40 mg lovastatin, 10 mg lisinopril, and 5 mg amlodipine)	no treatment	QALY	1- US$746–890/QALY (risk of CVD> 25%) 2- US$1039–1221/QALY (risk of CVD>5%)	DSA PSA	3%	3-GNI per capita	1–746–890 per QALY 2–1,360–1,599 per QALY
**Lim et al (2007) [38]**	low and middle income (23 countries)	Non	Primary & Secondary	Health care	A microsimulation model	10-years	SP: (aspirin, an angiotensin-converting-enzyme inhibitor, a β blocker, and a statin,) PP: (aspirin, an angiotensin-converting-enzyme inhibitor, thiazide and a statin)	no treatment	Number of deaths averted	US$ 2,625 per Death averted	PSA	NR	NR	3,329 per Death averted
**Newman et al (2008) [41]**	USA	CEA	primary	healthcare	Markov	10 years	polypill (simvastatin 40 mg, captopril 12.5 mg, hydrochlorothiazide 12.5 mg, and atenolol 25 mg)	current standard of caret	QALY	Dominant	DSA	3	$50,000/life-year threshold	Dominant
**Rubinstein et al (2009) [25]**	Argentina	CEA	primary	Payer	WHO-CHOICE	10 years	Polypill (thiazides 25 mg, enalapril 10 mg, atorvastatin 10 mg and aspirin 100 mg)	1- lowering salt intake 2- education through mass-media	DALY	$3,599(20% CVD risk), $4,113 (10% CVD risk) $4,533(5% CVD risk), per DALY averted	DSA	3%	3-GNP per capita	4,274 (20% CVD risk) 4,884 (10% CVD risk) 5383(5% CVD risk) per DALY averted
**Rubinstein et al (2010) [39]**	Argentina	CEA	primary	healthcare	NR	5-years	Polypill (hydrochlorothiazide 25 mg, enalapril 10 mg, atorvastatin 10 mg and aspirin 100 mg)	no treatment	DALY	Cost-saving	PSA Monte Carlo simulation	3%	GDP pre capita (US$ 6,644)	Cost-saving
**van Gils et al (2011) [42]**	Netherlands	CEA	primary	payer	Markov	lifetime	polypill (simvastatin 20 mg, thiazide 12.5 mg, ramipril 5 mg, atenolol 50 mg, aspirin100 mg)	Usual care	QALY YLs	€7200–10200 per LY €8500–12300 per QALY	PSA	0%, 1.5%, 3%, 4%, 5%	€20,000 Per LY or QALY	11,279–15,979 per LY 13,317–19,269 per QALY
**Khonputsa et al (2012) [43]**	Thailand	CEA	primary	healthcare	Markov	lifetime	Polypill (three BP-lowering drugs and a statin)	do-nothing	DALY	Dominant	PSA	3%	1–3 time GDP per-capita	Dominant
**Ito et al (2012) [44]**	USA	CEA	Secondary	societal	Markov	lifetime	Polypill (aspirin, a b-blocker, an ACEI or ARB, statin)	usual care, mailed education	QALY	Polypill:$133,000 polypill plus mailed education: $113,000 polypill plus disease management $142,900 per QALY	DSA	3%	$100,000 per QALY gained	Polypill: 151,160 polypill plus mailed education: 128,429 polypill plus disease managemen 162,412 per QALY
**Bautista et al (2013) [30]**	Latin America	CEA	Primary	Healthcare	Markov	lifetime	Polypill (thiazide, 12.5 mg; atenolol, 50 mg; ramipril, 5 mg, simvastatin, 20 mg, aspirin 100 mg)	do nothng	QALY	$158–804 per QALY in women 365–933 per QALY in men base on CVD risk	DSA	3%	GDP per Capita	176–895 per QALY in women 365–933 per QALY in men
**Zomer et al (2013) [45]**	Australia	CEA	Primary	Health care	Markov	10-years	polypill (three blood-pressure lowering, simvastatin, aspirin)	null scenario	YLs QALY	$301,583 per YLs $214,865 per QALY	PSA Monte Carlo simulation	5%	$50,000 per QALY or YLs	252,320 per YLs 179,767 per QALY
**Ong et al (2013) [46]**	Australia	CEA	primary	Health care	Markov	lifetime	Polypill (a statin, a diuretic, a beta blocker, a calcium channel blocker)	null scenario	DALY	Dominant	Monte Carlo simulation DSA	NR	$50,000 per DALY	Dominant
**Megiddo et al (2014) [47]**	India	CEA	Secondary	Healthcare	WHO-CHOICE	life time	Polypill (Aspirin, atorvastatin, ramipril, atenolol)	aspirin (75 mg once daily)	DALY	$1,690 per DALY averted	Latin hypercube sampling sensitivity analysis	3%	per capita GDP	1,854 per DALY averted
**Arrabal et al (2015) [48]**	Spain	CEA	Secondary	Healthcare	Markov	10-years	Polypill (100mg aspirin, 20mg atorvastatin and 10mg ramipril)	multiple monotherapy	QALY YLs	Dominant	PSA	Non	€ 30,000 per QALY gained	Dominant
**Becerra et al (2015) [28]**	UK	CEA	Secondary	Healthcare	Markov	10 years	Polypill (Aspirin 100 mg, atorvastatin 20 mg, ramipril 2.5, 5 or 10 mg)	multiple monotherapy	QALY YLs, adherence	£8200 per QALY	DSA PSA	3.5%	£20000–30000 per QALY gained	11,795 per QALY
**Wald et al (2016) [26]**	UK	CBA	primary	Healthcare	NR	life time	Polypill (20 mg simvastatin, 2.5 mg amlodipine, 25 mg losartan and 12.5 mg hydrochlorothiazide)	do nothing	the number of MIs and strokes and YLs gained without a first MI or stroke	If the cost per person per day were £0.56, a Polypill Prevention Programme would be cost neutral	DSA	NR	NR	If the cost per person per day were $0.81, a Polypill Prevention Programme would be cost neutral
**Barrios et al (2017) [27]**	Spain	CEA	Secondary	Healthcare	Markov	10-years	polypill (aspirin 100 mg, atorvastatin 20 mg, ramipril 10 mg)	multiple monotherapy	QALY LYs	Dominant	DSA PSA	3%	30,000 euros per QALY gained	Dominant
**Ferket et al (2017) [24]**	UK	CEA	Primary	Healthcare	microsimulation	life time	Polypill (statin & antihypertensive)	Current practice	QALY	£29,207 per QALY gained	PSA	3.50%	£20000–30000 per QALY gained	41,030 per QALY gained
**Jowett et al (2017) [49]**	UK	CUA	primary	Healthcare	Markov	10 years	polypill (40mg simvastatin, 12.5mg hydrochlorothiazide, 5mg lisinopril, 2.5mg amlodipine)	usual care (statin & antyhypertention)	QALY	Dominant up to £18,811 per QALY	DSA PSA	3.50%	£20000 per QALY	Dominant up to 26,426 per QALY
**Barth et al (2017) [50]**	Germany	CEA	secondary	Payer	Markov	life time	polypill (aspirin, simvastatin, lisinopril and atenolol)	Standard care	QALY	€ 9,228 per QALY	DSA	3%	NR	5,541 per QALY
**Singh et al (2018) [29]**	India	CEA	secondary	Healthcare	Within-trial cost-effectiveness analysis	15-month	polypill (aspirin, statin and two blood pressure lowering drugs)	usual care	increase in adherence reductions in SBP reductions LDL-c	Dominant	DSA	no discount	NR	Dominant
**Lin et al (2019) [51]**	China, India, Mexico, Nigeria, and South Africa	CEA	secondary	Healthcare	Markov	lifetime	polypill (aspirin 75 mg, lisinopril 10 mg, atenolol 50 mg, and simvastatin 40 mg)	current care	DALY	China:$168 India: $154 Mexico:$88 Nigeria: $364 South Africa: $64	DSA PSA	3%	GDP per Capita	China: 172 India: 169 Mexico: 90 Nigeria: 372 South Africa: 65
**Gaziano et al (2019) [52]**	USA	CEA	secondary	Healthcare Societal	CVD PREDICT micro-simulation model	5-years	aspirin 81 mg, atenolol 50mg, ramipril 5mg, and either simvastatin 40mg (Polypill I), atorvastatin 80 mg (Polypill II), or rosuvastatin 40 mg (Polypill III).	Usual care	QALY	Polypill I: 20,073 Polypill II: 20,571 Polypill III: 23,603	PSA	3%	$50000–150000 per QALY	Polypill I: 20,534 Polypill II: 21,043 Polypill III: 24,146
**Ntaios et al (2019) [53]**	Greece	CEA	secondary	Payer	Markov	life-time	Polypill		QALY	Dominant				Dominant

CEA: cost-effectiveness analysis; CUA: cost-utility analysis; CBA: cost-benefit analysis; QALY: quality-adjusted life year; DALY: disability-adjusted life year; YLs: years of life lost; DSA: deterministic sensitivity analyses; PSA: probabilistic sensitivity analyses; ICER: Incremental Cost-Effectiveness Ratio.

### Quality appraisal

The quality of the studies was assessed using CHEERS checklist. All studies included in this review met more than 50% of this checklist criteria. Based on the results, 15 articles had "excellent" quality (score above 85%), 6 articles classified as "very good" quality (score 70 to 85%), and 3 studies as "good" quality (score 50 to 70%). More recently published studies scored higher than the earlier studies. Details of the quality assessment are reported in Table 2.

**Table 2:** Quality appraisal of the included studies using the CHEERS checklist.

Study	Title	abstract	Introduction	Population characteristics	Setting and location	Study Perspective	Comparators described	Time horizon	Discount rate	Outcomes and relevance	Measurement of effectiveness	preference based outcomes	Costs	Currency, date and conversion	Model choice described	Model assumptions	Analysis methods	Parameters of values	Incremental costs	sensitivity analyses	Heterogeneity explained	Findings and limitations	Funding source	Potential conflict of interest	Total
Gaziano et al (2006) [6]	√	√	√	√	√	√	√	√	√	√	√	NA	√	×	√	√	√	√	√	√	√	√	√	√	92%
Franco et al (2006) [40]	#	√	√	√	#	√	√	√	√	#	√	NA	#	#	×	×	#	#	√	√	#	√	√	√	71%
Lim et al (2007) [38]	√	√	√	√	×	√	√	√	×	×	√	NA	√	×	×	×	√	×	×	×	√	√	×	√	54%
Newman et al (2008) [41]	√	√	√	√	√	√	√	√	√	√	√	NA	√	×	√	√	√	√	√	√	√	#	×	×	83%
Rubinstein et al (2009) [25]	√	√	√	√	√	√	√	√	√	√	√	×	√	×	√	√	√	√	√	√	√	√	√	√	91%
Rubinstein et al (2010) [39]	√	√	√	√	√	√	√	√	√	√	√	NA	√	√	×	√	√	√	√	√	#	√	√	√	89%
van Gils et al (2011) [42]	√	√	√	√	√	√	√	√	√	√	√	NA	√	×	√	√	√	√	√	√	√	√	√	√	92%
Khonputsa et al (2012) [43]	√	√	√	√	√	√	√	√	√	√	√	NA	√	×	√	√	√	√	√	√	√	√	√	√	92%
Ito et al (2012) [44]	√	√	√	√	√	√	√	√	√	√	√	NA	√	×	√	√	√	√	√	√	√	√	√	×	87%
Bautista (2013) [30]	√	√	√	√	√	√	√	√	√	√	√	NA	√	×	√	√	√	√	√	√	√	√	×	×	83%
Zomer et al (2013) [45]	√	√	√	√	√	√	√	√	√	√	√	NA	√	×	√	√	√	√	√	√	√	√	√	√	92%
Ong et al (2013) [46]	√	√	√	√	√	√	√	√	×	√	√	NA	√	×	√	√	√	√	√	√	√	√	√	×	83%
Megiddo et al (2014) [47]	√	√	√	√	√	√	√	√	√	√	√	NA	√	×	√	√	√	√	√	√	√	√	√	×	87%
Arrabal et al (2015) [48]	√	√	×	√	√	√	√	√	√	√	√	NA	√	×	√	√	√	√	√	√	√	√	×	×	79%
Becerra et al (2015) [28]	√	√	√	√	√	√	√	√	√	√	√	NA	√	×	√	√	√	√	√	√	√	√	√	√	92%
Wald et al (2016) [26]	√	√	√	√	√	√	√	√	NA	√	√	NA	√	NA	×	√	√	√	√	√	√	√	×	√	79%
Barrios et al (2017) [27]	√	√	√	√	√	√	√	√	√	√	√	NA	√	×	√	√	√	√	√	√	√	√	×	√	87%
Jowett et al (2017) [49]	√	√	√	√	√	√	√	√	√	√	√	NA	√	×	√	√	√	√	√	√	√	√	√	√	92%
Barth et al (2017) [50]	√	√	√	√	√	√	√	√	√	√	√	NA	√	√	√	√	√	√	√	√	#	√	√	√	94%
Ferket et al (2017) [24]	√	√	√	√	√	√	√	√	√	√	√	NA	√	×	√	√	√	√	√	√	√	√	√	√	92%
Singh et al (2018) [29]	√	√	√	√	√	√	√	√	NA	√	√	√	√	NA	NA	NA	NA	√	√	√	×	√	√	×	71%
Lin et al (2019) [51]	√	√	√	√	√	√	√	√	√	√	√	√	√	×	√	√	√	√	√	√	√	√	√	√	95%
Gaziano et al (2019) [52]	√	√	√	√	√	√	√	√	√	√	√	NA	√	×	√	√	√	√	√	√	√	√	√	×	87%
Ntaios et al (2019) [53]	√	√	×	√	√	√	√	√	√	√	√	NA	√	×	√	√	√	√	√	√	√	√	×	×	79%

√: Items that were completely met in the studies received a score of 1

#: items that were partially met in the studies received a score of 0.5

×: items that were not fulfilled at all received a score of zero, NA: Not Applicable

Aside from five studies, [27, 38, 41, 48, 53] the rest completely detail the financing condition for their initiatives. Twelve of them were backed by the government or research organizations, while the remaining eight were supported by industry [26, 28–30, 47, 51, 52]. Van Gils et al. [42] study did not receive any financial support. All studies, with the exception of seven [30, 41, 44, 46–48, 53], provided a conflict of interest statement, but none of them had a conflict of interest.

### Cost-effectiveness results

#### Primary prevention

In total, 14 studies have investigated the polypill as a primary prevention strategy. Ten studies [6, 24, 26, 30, 39, 41, 43, 45, 46, 49, 50] were analyzed from the perspective of the health care system.

The polypill in all studies contained at least one statin and two antihypertensive drugs. It contained aspirin in seven studies [6, 25, 30, 38, 39, 42, 45], and three antihypertensives in eight studies [24, 26, 30, 41–43, 45, 49]. The characteristics of the target population varied among the included studies. But, in general, healthy individuals with a high risk of CVD over the age of 30 years old, without any history of cardiovascular events were common characteristics of the population in all studies.

In 12 studies, the polypill was compared to "no therapy" (the absence of a comprehensive preventive program), with seven studies focusing on cost-effectiveness [6, 25, 26, 30, 38, 40, 43]. Polypill was dominant in two studies [41, 46], indicating that greater benefits may be obtained at a cheaper cost (i.e. health gain with cost-saving). In Zomer et al. [45] it wasn’t cost-effective. Polypill was not cost-effective in one scenario and totally dominated in four situations, according to Ferket et al [24] (i.e., more cost and less effect). Jowett et al. [47] and Van Gils [41] that compared polypill with usual care, identified it as a cost-effective intervention. Six studies had undertaken probabilistic sensitivity analyses [24, 38, 39, 42, 43, 45], four studies reported deterministic sensitivity analyses [25, 26, 30, 41] and four studies performed both of them. Furthermore, Rubinstein [39] and Lim et al. [38] included scenario analyses.

The price of polypill was identified as the main driver of cost-effectiveness in five studies [6, 24, 38, 46, 49]. Four studies [24, 30, 41, 49] considered the effect of age as the most influential parameter, and four studies [30, 38, 43, 46] reported the risks of CVD to be the key drivers of cost-effectiveness. Adherence to treatment [30, 38], drug efficacy [6, 41, 49], and drug combination [42, 43] were identified as other parameters affecting economic evaluation results.

#### Secondary prevention

As secondary prevention, polypill was examined in twelve studies. The majority of the research [6, 27, 29, 48, 50, 51] adopted the healthcare system viewpoint. Gaziano et al. [52] and Beccera et al. [28] have been analyzed from three and two perspectives respectively.

In total, the target population included adults aged over 30 years who have had at least one non-fatal coronary heart disease event and indication for secondary prevention treatment.

Polypill components, in all studies, included aspirin, a lipid-lowering agent, and at least one antihypertensive drug. Six studies contained two antihypertensive drugs [6, 29, 38, 42, 50–52].

Twelve studies compared polypill strategy with usual care (multiple monotherapies), of which in six studies, polypill was cost-effective [28, 38, 50–53]. In the other four studies, polypill was the dominant strategy [27, 29, 47, 48], that means it has been a more effective and cheaper strategy. Ito et al. [44], conclude that it was not cost-effective. In Gaziano et al. [6] where polypill is compared with no treatment, this strategy was cost-effective.

Studies have done deterministic [29, 44, 50], probabilistic [38, 48, 52, 53] sensitivity analysis or both of them [6, 27, 28, 51]. Megiddo et al. [47] measured their results with Latin hypercube sampling sensitivity analysis. Different factors drive cost effectiveness. Polypill price was the most important driver in the four studies [6, 29, 38, 44]. Adherence to treatment was identified in five studies [28, 29, 38, 44, 52]. Besides, utility [27, 28] and CVD risk [28, 38] were the other key drivers of ICER. Furthermore, in Becerra et al [28] and Barrios et al [27] the ICER was sensitive to the discount rate.

## Discussion

This systematic review summarized 24 published economic evaluations of polypill in the prevention of CVD. Most of the included studies had high methodologic quality. Except for one, all of the research assessed cost-effectiveness, with the majority of them focusing on healthcare cost. The cost of polypill was regarded as one of the most important cost-effectiveness factors. There were two types of studies: primary and secondary prevention. In 14 of the 24 studies, primary prevention was the focus, with polypill proving to be a cost-effective or cost-saving technique in 10 of them. Although Zomer et al. [45], concluded that the polypill wasn’t cost-effective, they stated that, in high-risk populations, it may be cost-effective compared to using antihypertensive alone or antihypertensive plus statins. Furthermore, Ferket et al. [24] indicated that beginning polypill at the age of 60 and lowering the yearly cost of polypill to less than £ 240 and £60 correspondingly makes it a cost-effective and cost-saving alternative. Furthermore, Rubinstein et al [25] discovered that using polypill in combination with salt reduction and health education to target persons at a 20% or higher risk was cost-effective. The research by Jowett et al. [49] further brings out that it is a cost-effective intervention for persons over the age of 50.

As secondary prevention in all but one study, polypill was a cost-effective approach compared to usual care. In Ito et al. [44], polypill particularly when combined with mailed educational materials, could be cost-effective, and potentially cost-saving if its price decreased to less than $100 per month. Polypill following Ezetimibe and omega-3 polyunsaturated fatty acids (n-3 PUFAs) were regarded as cost-effective techniques in secondary prevention of CVD in a recent systematic review [54]. However, the mentioned systematic review only looked at novel strategies in secondary prevention settings, and only included seven trials, leaving out the other studies considered in the present study.

Significant differences in ICER values among studies (ranging from 24 to 31000 dollars in adjusted 2020 US dollars) are due to a wide range of treatment patterns and healthcare system structures, as well as differences in the delivery and cost of healthcare services and reimbursement mechanisms available in different countries. Furthermore, research analyzed data from various viewpoints, temporal periods, and model assumptions. Furthermore, the threshold, defined as the relative value against which acceptability is measured, ranged from $6644 in Argentina [39] to 150 thousand dollars in the United States [52]. As a consequence, comparing and generalizing the outcomes of these economic analyses should be done with care.

The present systematic review has identified several of challenges in included studies. First, clinical trial studies emphasized adherence improvement as one of the main advantages of polypill compared to multiple monotherapies in the prevention of CVD [55]. As well, World Health Organization recommended that improving adherence to treatment may have a greater impact on the health of the population than any new intervention [40]. But, only a few studies have considered the relative increase in the treatment adherence in modeling. That’s why, the cost-effectiveness of polypill may be underestimated.

Second, the number, type and dosage of drugs used in the composition of polypill are different among the studies, which can lead to different intermediate (cholesterol and blood pressure) and final (CVD events or mortality) outcomes. Subsequently, it may affect the results of cost effectiveness analysis.

Third, the price of polypill is one of the key drivers of cost-effectiveness [6, 24, 29, 38, 44, 46, 49].

Polypill is a fixed dose combination of several drugs, so in studies its price has been assumed to be equal to or greater than the sum of the individual medication prices.

However, according to sensitivity analysis results, cutting the price of a polypill makes it more cost-effective or even cost-saving when compared to standard treatment.

Fourth, cardiovascular disease imposes substantial related productivity loss costs due to absenteeism, presenteeism, early retirement, and premature mortality, especially in low- and middle-income countries [56, 57]. Despite the fact that integrating productivity losses on the ICER improves cost-effectiveness [58], only two research [44, 52] took a societal viewpoint into account. After converting from a healthcare to a social viewpoint, all three polypill solutions were cost-effective when compared to standard treatment, according to Gazianio et al. [52]. In addition, Ito et al. [44] include just the expenses of long-term care and informal care as indirect costs, and the cost of lost production was not considered.

Fifth, in addition to the many benefits of a polypill-based strategy, there are potential concerns about decreased medication choice, limited flexibility in dose titration, the impact of drug intolerance, low physician acceptability, and mass medicalization, which have not been considered in studies [59].

Sixth, prevention strategies, such as promoting changes in nutritional habits, physical activity, alcohol consumption, weight, and smoking in CVD are diverse, and they could be equally or more cost-effective than the polypill, especially in primary prevention. For example, Rubinstein et al. studies [25, 39] showed that less salt in bread and mass media campaign is more cost-effective than the polypill. However, due to the lack of data on the effectiveness and cost of these strategies, they have not been imported into the models.

Researchers provided several solutions to address these obstacles and get a better understanding of the cost-effectiveness of the polypill in the preventative context of cardiovascular disease. Using the results of long-term clinical trial studies with a larger patient population like TIPS-3 [60], SECURE [61], and PolyIran [21] trials are expected to provide further insight into the efficacy as well as the improvement in adherence and are needed to confirm the advantages of this approach over multiple monotherapies and tease out the difference between these two approaches in the future cost-effectiveness studies. Given the concerns expressed, it is necessary to pay attention to the customization of the different polypill into various indications such as coronary heart disease (CHD), stroke, Myocardial Infarction, and high-risk primary prevention.

Furthermore, it is necessary to investigate the polypill, particularly as primary prevention, in comparison to other preventive measures such as regular physical activity, healthy diet, and maintaining healthy body weight to determine their respective roles in preventing cardiovascular events. Furthermore, generic dosage forms are used to manufacture polypills, which minimize packaging, distribution, and marketing expenses, as well as the frequency of doctor visits and laboratory tests, lowering the price of polypills and treatment costs. Hence, it is predicted that the price of polypill under these circumstances will cost around $ 1 per day in high-income countries and less than 20 cents per day in developing countries [62]. As a result, affordability and availability will improve. Subsequent economic evaluation studies should also be considered from a social perspective, to provide more insight to policymakers in integrating this approach with other approaches.

This systematic review has several strengths. It is the first study to review the cost-effectiveness of the polypill in both primary and secondary prevention levels in CVDs. This review follows the Preferred Reporting Items for Systematic Reviews and Meta-Analyses guideline. To minimize the risk of missing relevant studies, in addition to major databases, supplemental searches including the bibliographies of all included studies and grey literature were searched and no language restrictions have been imposed.

There are two limitations to this review. First, pooling the findings was infeasible owing to methodological, clinical, and healthcare environment incompatibility amongst research. Second, this review focuses on full economic evaluation studies (CEA, CBA, and CUA), and partial economic evaluation studies such as cost analyses were excluded. Full economic evaluation is the comparative analysis of alternative courses of action in terms of both costs (resource use) and consequences (outcomes, effects) while focus on costs and resource use, or partial economic evaluation and can contribute useful evidence to an understanding of economic aspects of interventions.

## Conclusion

The polypill seems to be a cost-effective way to enhance outcomes in primary and secondary prevention of CVD, according to this systematic analysis. Because the applicability of cost-effectiveness findings is debatable, further economic assessment studies based on long-term clinical trials with large populations are required. When extending the findings to their nation, policymakers should be mindful of how the polypill interacts with other primary and secondary preventive strategies as a complementary health strategy.

## Supporting information

S1 Table PRISMA checklist. (DOCX)

S2 Table Search strategies. (DOCX)
