# Correction: Cost-effectiveness of fixed-dose combination pill (Polypill) in primary and secondary prevention of cardiovascular disease: A systematic literature review

**DOI:** 10.1371/journal.pone.0293219

**Published:** 2023-10-18

**Authors:** Reza Jahangiri, Aziz Rezapour, Reza Malekzadeh, Alireza Olyaeemanesh, Gholamreza Roshandel, Seyed Abbas Motevalian

There are errors in the Funding section. The correct Funding statement is: This article is part of the PhD dissertation approved at Iran University of Medical Sciences (Grant No. IUMS/SHMIS_98-3-37-15974, Ethical Code. IR.IUMS.REC.1398.589). The funders had no role in study design, data collection and analysis, decision to publish, or preparation of the manuscript.
