# Supplementary material for: Cost-effectiveness of fixed-dose combination pill (Polypill) in primary and secondary prevention of cardiovascular disease: A systematic literature review
Source: PLoS One. 2022 Jul 28;17(7):e0271908. doi: 10.1371/journal.pone.0271908 (PMC9333258; doi:10.1371/journal.pone.0271908)
Supplement: S2 Table — (DOCX) [file pone.0271908.s002.docx]

### Supporting information

### S2 table. Search Strategies

| **Database** | **Search Strategy** | **NO** |
| --- | --- | --- |
| **PUBMED/**  **MEDLINE** | - #1- (”Multidrug”[Title/Abstract] OR ”Fixed dose combination”[Title/Abstract] OR ” polypill”[Title/Abstract]) - #2- (”Cost benefit”[Title/Abstract] OR economic*[Title/Abstract] OR cost[Title/Abstract] OR ”cost-effectiveness”[Title/Abstract] OR ”Cost utility”[ Title/Abstract] OR ” Economic evaluation”[Title/Abstract] OR ”Models, Economic”[Mesh]) - #3- (prevention*[Title/Abstract] OR ”primary prevention”[Title/Abstract] OR ” secondary prevention”[Title/Abstract] OR ”Cardiovascular disease”[Title/Abstract] OR ”heart disease”[Title/Abstract] OR ” Myocardial infraction”[ Title/Abstract]) - #1 AND #2 AND #3 | **325** |
| **WEB OF SCIENCE** | - #1- TS=(("Multidrug" OR "Fixed dose combination" OR "Polypill")) Indexes=SCI-EXPANDED, SSCI, A&HCI, CPCI-S, CPCI-SSH, BKCI-S, BKCI-SSH, ESCI Timespan=2003-2020 - #2- TS=((economic* OR cost OR "Cost-benefit" OR "cost-effectiveness" OR "Cost-utility" OR "Economic evaluation")) Indexes=SCI-EXPANDED, SSCI, A&HCI, CPCI-S, CPCI-SSH, BKCI-S, BKCI-SSH, ESCI Timespan=2002-2020 - #3- TS=("Cardiovascular disease" OR "heart disease" OR "Myocardial infraction") Indexes=SCI-EXPANDED, SSCI, A&HCI, CPCI-S, CPCI-SSH, BKCI-S, BKCI-SSH, ESCI Timespan=2003-2020 - #4- TS=(prevention* OR "primary prevention" OR "secondary prevention") Indexes=SCI-EXPANDED, SSCI, A&HCI, CPCI-S, CPCI-SSH, BKCI-S, BKCI-SSH, ESCI Timespan=2003-2020 - #1 AND #2 AND #3 | **111** |
| **SCOPUS** | - #1- TITLE-ABS-KEY ( multidrug OR "fixed dose combination" OR polypill) - #2- TITLE-ABS-KEY ( "Cost benefit" OR "cost effectiveness" OR "Cost utility" OR "Economic evaluation" ) - OR TITLE-ABS-KEY (Cost-utility) OR TITLE-ABS-KEY (Economic evaluation)) - #3- TITLE-ABS-KEY (prevention OR "primary prevention" OR "secondary prevention") - #4- TITLE-ABS-KEY ("cardiovascular disease" OR "heart disease" OR "myocardial infraction") - #1 AND #2 AND #3 AND #4 | **92** |
| **COCHRANE** | - #1- multidrug OR "fixed dose combination" OR polypill in Title Abstract Keyword - #2- "Cost benefit" OR "cost effectiveness" OR "Cost utility" OR "Economic evaluation" in Title Abstract Keyword - 3#- prevention OR "primary prevention" OR "secondary prevention" in Title Abstract Keyword - #4- "cardiovascular disease" OR "heart disease" OR "myocardial infraction" in Title Abstract Keyword - #1 AND #2 AND #3 AND #4 | **14** |
| **EMBASE** | - #1- (”Multidrug”:ab,ti OR ”Fixed dose combination”:ab,ti OR ” Polypill”:ab,ti) - #2- (”economic*:ab,ti OR cost:ab,ti OR ”Cost benefit”:ab,ti OR ”cost-effectiveness”:ab,ti OR ”Cost utility”[ Title/Abstract] OR ”Economic evaluation”:ab,ti OR ”economic evaluation”/exp) - #3- (prevention*:ab,ti OR ”primary prevention”:ab,ti OR ” secondary prevention”:ab,ti OR ”Cardiovascular disease”:ab,ti OR ”heart disease”:ab,ti OR ” Myocardial infraction/exp) - #1 AND #2 AND #3 | **147** |
| **EconLit (EBSCO)** | - #1- (ti,ab(Multidrug) OR ti,ab(Fixed dose combination) OR ti,ab(Polypill))      - #2- (ti,ab(economic*) OR ti,ab(cost) OR ti,ab(Cost-benefit) OR ti,ab(cost-effectiveness) OR ti,ab(Cost-utility) OR ti,ab(Economic evaluation)) - #3- (ti,ab(prevention*) OR ti,ab(primary prevention) OR ti,ab(secondary prevention) OR ti,ab(Cardiovascular disease) OR ti,ab(heart disease) OR ti,ab(Myocardial infraction)) - #1 AND #2 AND #3 | **87** |
| **CINAHL** | - #1 TX (”Multidrug” OR ”Fixed dose combination” OR ”polypill”) - #2 TX (”Cost-benefit” OR ”cost-effectiveness” OR ”Cost-utility” OR “Economic evaluation”) - #3 TX (”prevention” OR ”primary prevention” OR ”secondary prevention”) - #4 TX (”Cardiovascular disease” OR ”heart disease” OR “Myocardial infraction”) - #1 AND #2 AND #3 AND #4 | **138** |
